# Supplementary figures and images for: Social perception of mesocarnivores within hunting areas differs from actual species abundance
Source: PLoS One. 2023 Apr 26;18(4):e0283882. doi: 10.1371/journal.pone.0283882 (PMC10132647; doi:10.1371/journal.pone.0283882)

A

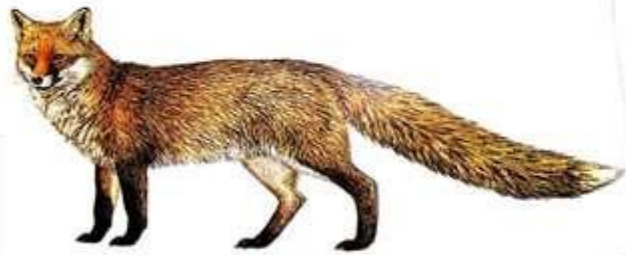

1

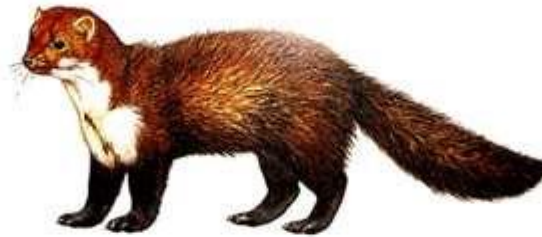

2

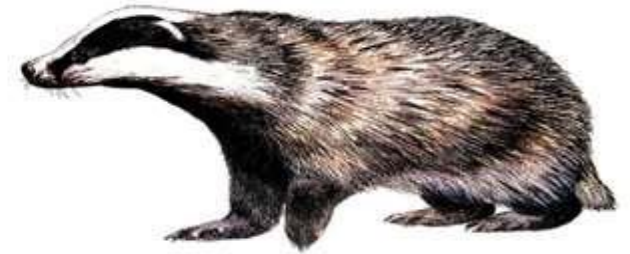

3

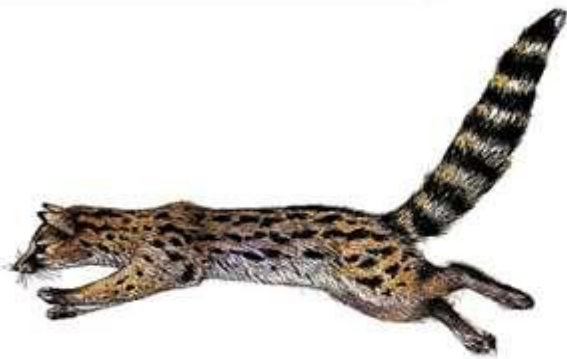

4

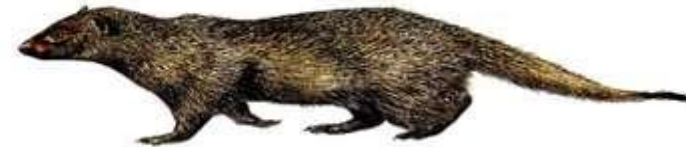

5

**B**

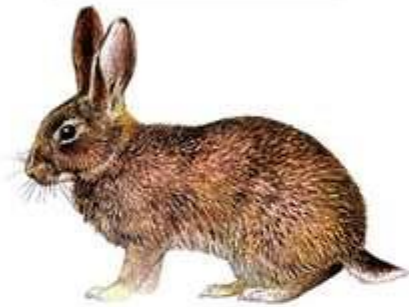

Conejo

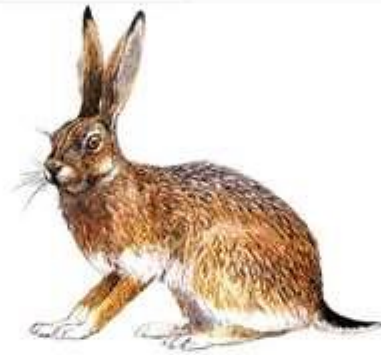

Liebre

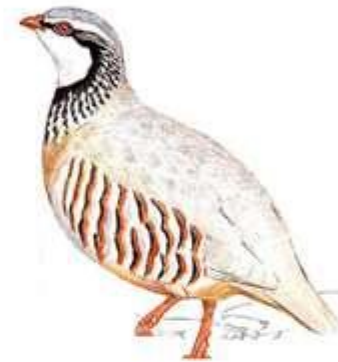

Perdiz

Supplement: S1 Fig — Plates with color images employed during the surveys representing A) the mesocarnivore species, and B) the small game species, considered in the study. 1) Red fox (Vulpes vulpes); 2) Stone marten (Martes foina); 3) Eurasian badger (Meles meles); common genet (Genetta genetta); 5) Egyptian mongoose (Herpestes ichneumon). (PDF) [file pone.0283882.s001.pdf]

A

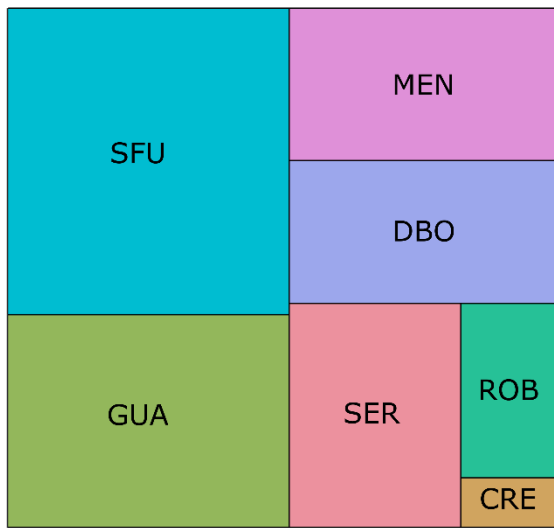

B

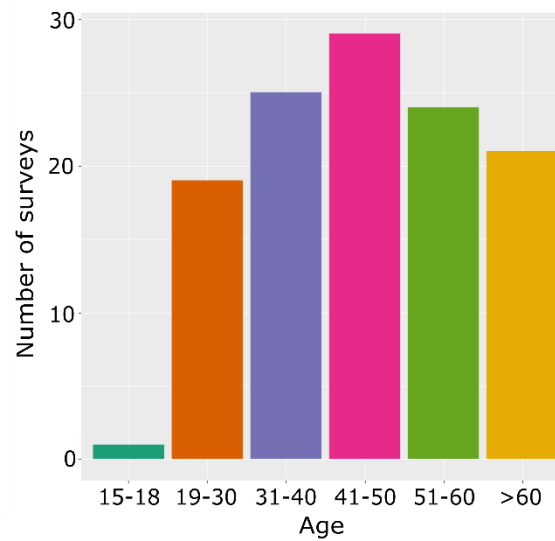

C

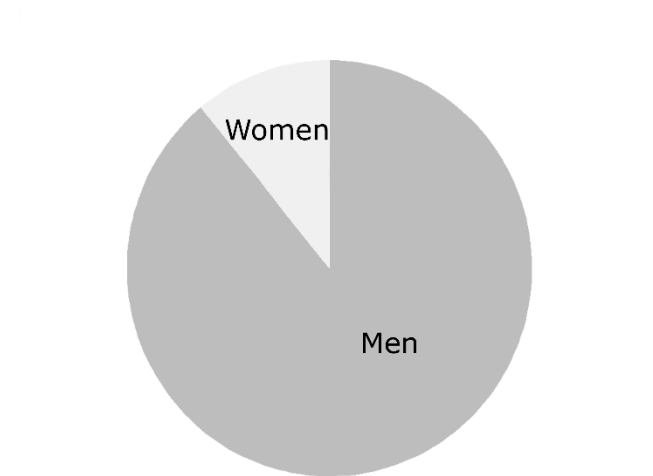

D

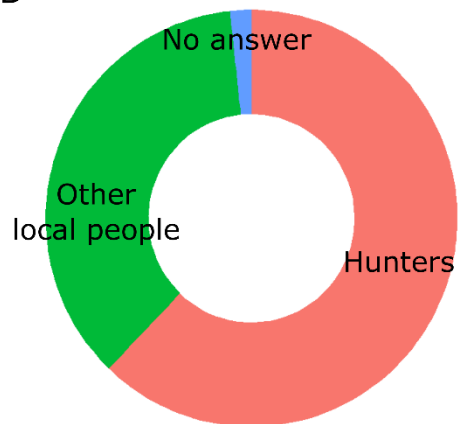

E

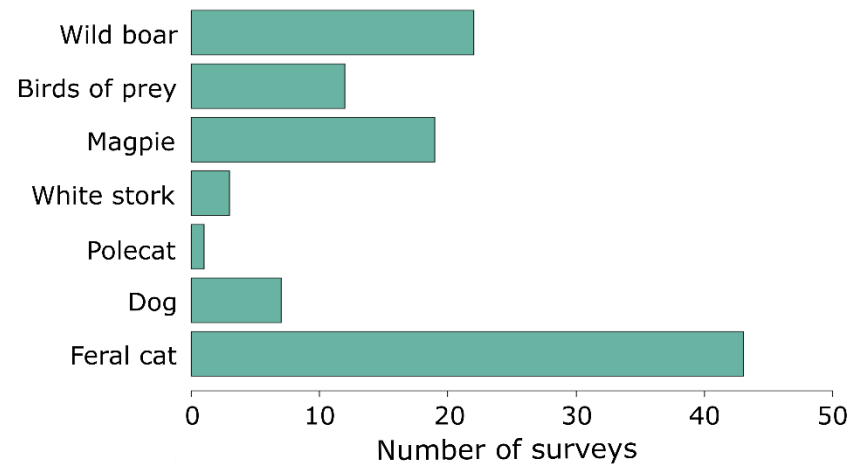

Supplement: S2 Fig — A) Proportion of surveys carried out in each hunting area. B) Number of surveys completed by each age group. C) Proportion of surveys completed regarding the gender of the respondent. D) Proportion of surveys completed by hunters and other local people. E) Number of surveys in which respondents considered additional species that need to be controlled. (PDF) [file pone.0283882.s002.pdf]

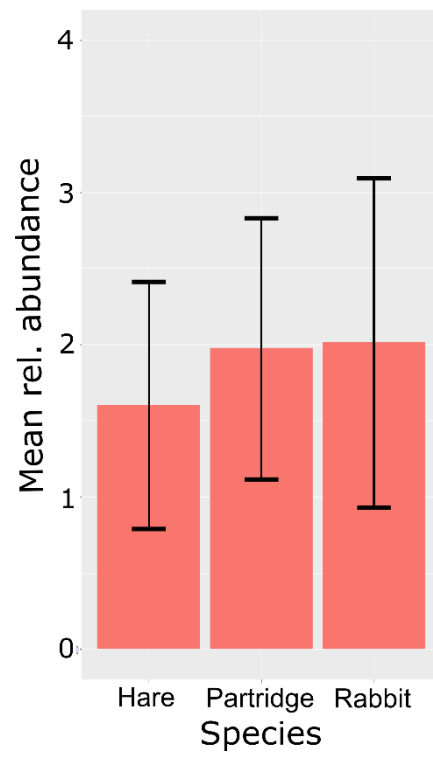

Supplement: S3 Fig — Mean relative abundance of small game species perceived by the respondents. (PDF) [file pone.0283882.s003.pdf]

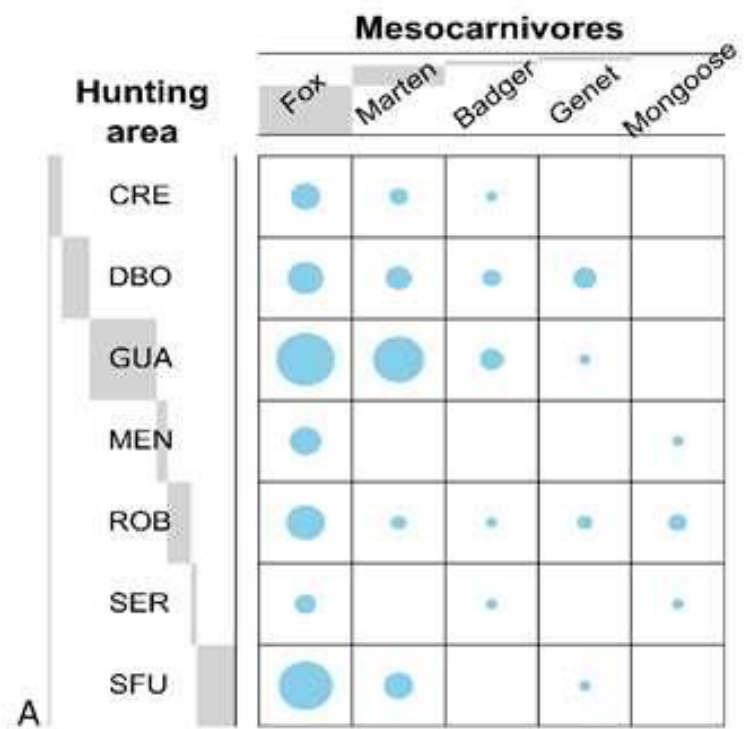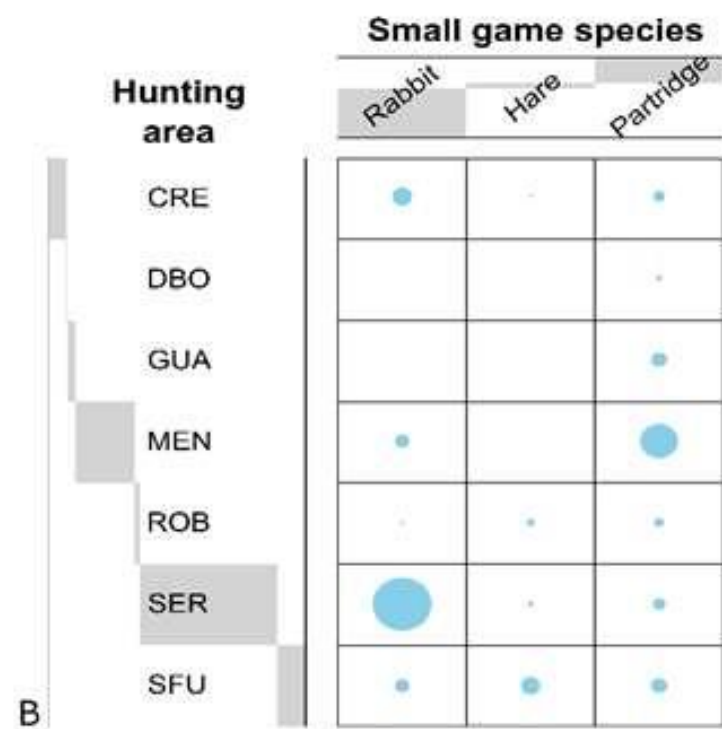

Supplement: S4 Fig — Relative abundance of A) mesocarnivores, and B) small game species, recorded during field samplings. Grey bars represent the proportion of records attributed to the species registered (in columns) and the proportion of records found across each hunting area (in rows). Light blue dots represent the relative abundance of each species across each hunting area. Differences in dot size represent between-species differences in relative abundance. CRE: Cabeza Redonda; DBO: Dehesa Boyal; GUA: Guadalupe: MEN: Mengabril; ROB: Robledo; SER: Serrezuela 3; SFU: Sierra de Fuentes. (PDF) [file pone.0283882.s004.pdf]
