# Supplementary material for: Social perception of mesocarnivores within hunting areas differs from actual species abundance
Source: PLoS One. 2023 Apr 26;18(4):e0283882. doi: 10.1371/journal.pone.0283882 (PMC10132647; doi:10.1371/journal.pone.0283882)
Supplement: S1 Table — (PDF) [file pone.0283882.s005.pdf]

		Stone marten			Eurasian badger			Common genet			Egyptian mongoose		
		**Coefficient**	**SE**	**P**	**Coefficient**	**SE**	**P**	**Coefficient**	**SE**	**P**	**Coefficient**	**SE**	**P**
A)	*Medium-low*	**1.02**	**0.50**	**0.04**	1.38	0.82	0.09	1.02	0.69	0.14	0.92	0.87	0.29
	Medium-high	**2.80**	**1.11**	**0.01**	1.48	1.09	0.17	0.23	0.73	0.75	**2.17**	**0.82**	**0.01**
	High	17.73	2065.95	0.99	15.90	1508.85	0.99	0.97	1.11	0.38	**4.28**	**0.93**	**0.00**
B)	*Medium-low*	1.08	0.57	0.06	0.03	0.67	0.97	0.28	0.62	0.66	-15.59	1659.44	0.99
	Medium-high	0.63	0.65	0.33	1.72	1.09	0.11	0.00	0.76	1.00	1.32	0.90	0.14
	High	0.63	0.58	0.28	16.23	1377.73	0.99	0.24	0.67	0.72	**2.36**	**0.68**	**0.00**

Red fox was excluded from the analysis due to the high rate of successful identification shown by the respondents (97.48%)
