# Supplementary material for: Social perception of mesocarnivores within hunting areas differs from actual species abundance
Source: PLoS One. 2023 Apr 26;18(4):e0283882. doi: 10.1371/journal.pone.0283882 (PMC10132647; doi:10.1371/journal.pone.0283882)
Supplement: S2 Table — Parameter estimates showing differences in the perception of A) mesocarnivore relative abundance, and B) damage caused to small game species, between hunters and other local people. (PDF) [file pone.0283882.s006.pdf]

	Red fox			Stone marten			Eurasian badger			Common genet			Egyptian mongoose		
	**Coefficient**	**SE**	**P**	**Coefficient**	**SE**	**P**	**Coefficient**	**SE**	**P**	**Coefficient**	**SE**	**P**	**Coefficient**	**SE**	**P**
**19-30**				**-2.52**	**1.21**	**0.04**	-0.52	0.90	0.56	-1.35	0.90	0.13	0.00	0.92	1.00
**31-40**				-0.59	0.95	0.53				-0.50	0.97	0.60	0.83	1.08	0.45
**41-50**	-1.70	1.79	0.34	-0.96	0.87	0.27	1.36	1.01	0.18	-0.68	0.87	0.43	0.33	0.88	0.71
**51-60**	-1.84	1.57	0.91	-0.22	0.80	0.79	0.04	0.81	0.96	-0.06	0.85	0.94	-0.66	0.81	0.42

The group ‘>60’ is included in the intercept. Significant differences between hunters and other local people are marked in bold
