# Supplementary material for: Social perception of mesocarnivores within hunting areas differs from actual species abundance
Source: PLoS One. 2023 Apr 26;18(4):e0283882. doi: 10.1371/journal.pone.0283882 (PMC10132647; doi:10.1371/journal.pone.0283882)
Supplement: S3 Table — Parameter estimates showing differences in the perception of A) mesocarnivore relative abundance, and B) damage caused to small game species, depending on the ability to adequately identify the species. (PDF) [file pone.0283882.s007.pdf]

		Red fox			Stone marten			Eurasian badger			Common genet			Egyptian mongoose		
		**Coefficient**	**SE**	**P**	**Coefficient**	**SE**	**P**	**Coefficient**	**SE**	**P**	**Coefficient**	**SE**	**P**	**Coefficient**	**SE**	**P**
A)	*Medium-low*	-2.18	2.61	0.40	0.89	0.58	0.12	**1.68**	**0.64**	**0.01**	**1.63**	**0.67**	**0.02**	0.92	1.03	0.37
	Medium-high	-0.11	1.41	0.94	1.84	1.20	0.13	**1.76**	**0.76**	**0.02**	0.43	0.79	0.59	**2.06**	**0.86**	**0.02**
	High	1.40	1.25	0.26	0.46	1.49	0.76	1.19	0.87	0.17	1.35	1.33	0.31	**2.54**	**0.78**	**>0.01**
B)	*Medium-low*	0.75	1.44	0.60	**1.85**	**0.81**	**0.02**	1.23	0.69	0.08	**1.43**	**0.66**	**0.03**	1.54	1.50	0.31
	Medium-high	**5.97**	**1.72**	**<0.01**	**2.05**	**0.91**	**0.02**	**1.46**	**0.71**	**0.04**	1.09	0.82	0.18	1.17	1.07	0.27
	High	**5.45**	**1.55**	**<0.01**	**1.65**	**0.80**	**0.04**	1.26	0.68	0.07	**1.69**	**0.85**	**0.05**	**3.04**	**0.88**	**<0.01**

The group ‘other local people’ and the ‘Low’ value of relative abundance and damage caused to small game species are included in the intercept. Significant differences are marked in bold.
