# Supplementary material for: Social perception of mesocarnivores within hunting areas differs from actual species abundance
Source: PLoS One. 2023 Apr 26;18(4):e0283882. doi: 10.1371/journal.pone.0283882 (PMC10132647; doi:10.1371/journal.pone.0283882)
Supplement: S4 Table — (PDF) [file pone.0283882.s008.pdf]

	European rabbit			Iberian hare			Red-legged partridge		
	**Coefficient**	**SE**	**P**	**Coefficient**	**SE**	**P**	**Coefficient**	**SE**	**P**
Medium-low	-0.23	0.78	0.77	-0.56	0.53	0.29	**1.41**	**0.61**	**0.02**
Medium-high	-0.69	0.78	0.38	-0.94	0.88	0.29	0.28	0.64	0.67
High	-1.73	1.00	0.08				-0.16	3.82	0.97

The group 'other local people' and the 'Low' value of relative abundance are included in the intercept. Given that the Iberian hare was considered highly abundant only by a minority of respondents (4.20%), model output for the 'High' value of relative abundance was omitted. Significant differences are marked in bold.
