# Supplementary material for: Social perception of mesocarnivores within hunting areas differs from actual species abundance
Source: PLoS One. 2023 Apr 26;18(4):e0283882. doi: 10.1371/journal.pone.0283882 (PMC10132647; doi:10.1371/journal.pone.0283882)
Supplement: S5 Table — Parameter estimates showing differences in the perception of A) mesocarnivore relative abundance, and B) damage caused to small game species, between age groups. (PDF) [file pone.0283882.s009.pdf]

			Mesocarnivores											
			Stone marten			Eurasian badger			Common genet			Egyptian mongoose		
			**Coefficient**	**SE**	**P**	**Coefficient**	**SE**	**P**	**Coefficient**	**SE**	**P**	**Coefficient**	**SE**	**P**
**A)**		*Medium-low*	0.41	0.91	0.65	0.44	1.05	0.68	-0.77	1.02	0.45	-0.19	1.64	0.91
	**19-30**	*Medium-high*	1.72	1.65	0.30	1.94	1.50	0.20				0.18	1.50	0.91
		High				0.38	1.52	0.80				1.38	1.39	0.32
		Medium-low	0.10	0.84	0.91	1.43	0.95	0.13	-0.44	92.00	0.64			
	**31-40**	*Medium-high*				**3.28**	**1.46**	**0.02**	-2.23	1.35	0.10			
		High												
		Medium-low	-0.16	0.86	0.86	0.92	0.90	0.30	-1.01	0.95	0.29	-3.28	1.89	0.08
	**41-50**	*Medium-high*	1.21	1.39	0.38	1.25	1.46	0.39	-2.49	1.31	0.06	-0.92	1.24	0.46
		High	-1.03	1.29	0.42	0.88	1.06	0.41	78.00	1.13	0.49	0.91	1.04	0.38
		Medium-low	0.54	0.79	0.50	0.61	0.83	0.46	0.04	0.90	0.97	-2.72	1.51	0.07
	**51-60**	*Medium-high*	1.62	1.26	0.20	1.66	1.27	0.19	-0.80	0.95	0.40	-1.81	1.18	0.13
		High				-1.78	1.26	0.16	-1.93	1.37	0.16	0.07	0.88	0.94
**B)**		*Medium-low*	1.06	1.33	0.43	-0.38	1.14	0.74	0.22	0.99	0.83	-0.68	1.79	0.70
	**19-30**	*Medium-high*	1.21	1.00	0.23	-0.07	1.02	0.95	-0.39	1.36	0.78	-0.54	1.35	0.69
		High	0.34	1.12	0.76	-0.24	1.08	0.83	1.21	1.14	0.29	-0.25	1.13	0.82
		Medium-low	1.58	1.17	0.18	-0.26	1.01	0.80	1.05	0.89	0.24			
	**31-40**	*Medium-high*				-1.43	1.29	0.27	-0.51	1.32	0.70	0.57	1.49	0.70
		High	0.18	1.00	0.86	0.62	0.93	0.48	0.98	1.10	0.37	0.63	1.32	0.63
		Medium-low	0.70	1.13	0.54	0.08	0.92	0.93	0.19	0.84	0.82	-0.53	1.78	0.77
	**41-50**	*Medium-high*	-1.06	1.27	0.41	-0.02	0.87	0.98	-0.51	1.06	0.63	-1.55	1.57	0.32
		High	-0.27	0.95	0.78	-0.57	0.98	0.57	0.43	0.97	0.66	0.48	1.09	0.66
		Medium-low	1.45	1.11	0.19	-0.02	0.89	0.98	0.05	0.93	0.95			
	**51-60**	*Medium-high*	0.60	0.92	0.52	-0.66	0.86	0.44	0.52	0.96	0.58			
		High	-0.03	0.93	0.98	-1.31	0.99	0.19	0.97	0.95	0.31	-0.36	0.95	0.71

The group '>60' and the 'Low' value of relative abundance and damage caused to small game species are included in the intercept. Significant differences are marked in bold.

Blank cells represent values of relative abundance and/or damage caused to small game species for which models did not converge due to small sample size.
