# Supplementary material for: Social perception of mesocarnivores within hunting areas differs from actual species abundance
Source: PLoS One. 2023 Apr 26;18(4):e0283882. doi: 10.1371/journal.pone.0283882 (PMC10132647; doi:10.1371/journal.pone.0283882)
Supplement: S6 Table — (PDF) [file pone.0283882.s010.pdf]

		European rabbit			Iberian hare			Red-legged partridge		
		**Small game species**								
		**Coefficient**	**SE**	**P**	**Coefficient**	**SE**	**P**	**Coefficient**	**SE**	**P**
**19-30**	*Medium-low*	-0.15	1.36	0.91	0.23	0.86	0.79	-0.98	1.01	0.33
	Medium-high	0.36	1.31	0.78				0.56	1.07	0.60
	High	0.74	1.44	0.61	-0.22	1.57	0.89			
**31-40**	*Medium-low*	1.75	1.19	0.14	1.07	0.76	0.16	-0.77	0.90	0.39
	Medium-high	1.68	1.38	0.22				-0.11	1.01	0.91
	High	0.72	1.56	0.64				-0.29	1.43	0.84
**41-50**	*Medium-low*	1.30	1.08	0.23	1.25	0.73	0.09	-0.18	0.88	0.84
	Medium-high	0.00	1.27	1.00				0.37	0.99	0.71
	High	-2.31	1.76	0.19	0.18	1.54	0.91	-0.96	1.62	0.55
**51-60**	*Medium-low*	1.19	1.09	0.28	0.24	0.76	0.75	-1.68	0.90	0.06
	Medium-high	1.37	1.16	0.24				-0.32	0.97	0.74
	High	0.69	1.26	0.58	-0.16	1.50	0.92			

The group '>60' and the 'Low' value of relative abundance are included in the intercept. Blank cells represent values of relative abundance for which models did not converge due to small sample size.
