# Supplementary material for: Social perception of mesocarnivores within hunting areas differs from actual species abundance
Source: PLoS One. 2023 Apr 26;18(4):e0283882. doi: 10.1371/journal.pone.0283882 (PMC10132647; doi:10.1371/journal.pone.0283882)
Supplement: S7 Table — Degree of similarity between the relative abundance perceived by A) the total of respondents, B) hunters, and C) Other local people and the relative abundance registered during the field samplings. (PDF) [file pone.0283882.s011.pdf]

Mesocarnivores

A)	Fox	Marten	Badger	Genet	Mongoose
CRE	3	0	0	0	1
DBO	2	1	0	1	2
GUA	0	1	1	2	3
MEN	2	1	0	1	2
ROB	2	1	2	1	0
SER	3	1	1	1	2
SFU	0	1	2	1	2
B)					
CRE					
DBO	3	2	3	2	3
GUA	2	2	0	1	3
MEN	0	2	2	1	3
ROB	2	1	2	2	2
SER	2	0	1	0	0
SFU	3	1	2	1	2
C)					
CRE	3	0	0	0	1
DBO	2	3	1	1	2
GUA	2	0	0	2	0
MEN	1	0	0	0	1
ROB					
SER	3	1	1	0	2
SFU	0	0	1	1	2

Small game

A)	Rabbit	Hare	Partridge
CRE	2	0	3
DBO	1	1	1
GUA	1	1	2
MEN	1	1	1
ROB	2	1	2
SER	0	1	2
SFU	2	2	1
B)			
CRE			
DBO	1	1	1
GUA	1	1	2
MEN	1	1	1
ROB	2	1	2
SER	0	1	1
SFU	2	2	1
C)			
CRE	2	0	3
DBO	2	1	1
GUA	0	0	1
MEN	0	0	3
ROB			
SER	0	1	2
SFU	2	1	1

Cell colour intensity and value represent the degree of similarity between the relative abundance perceived by the respondents and the relative abundance registered during the field samplings. Specifically, the highest the degree of similarity, the lowest the colour intensity and the value of the cells, and vice versa. Blank cells represent hunting areas in which one of the surveyed groups (i. e. hunters and other local people) was not represented by any respondent
