# Supplementary material for: Social perception of mesocarnivores within hunting areas differs from actual species abundance
Source: PLoS One. 2023 Apr 26;18(4):e0283882. doi: 10.1371/journal.pone.0283882 (PMC10132647; doi:10.1371/journal.pone.0283882)
Supplement: S8 Table — (PDF) [file pone.0283882.s012.pdf]

Tracks

Hunting area	Mesocarnivores					Small game species		
	Red fox	Stone marten	Eurasian badger	Common genet	Egyptian mongoose	European rabbit	Iberian hare	Red-legged partridge
Cabeza Redonda	**1**	**0**	**1**	**0**	**0**	**2**	**1**	**16**
Dehesa Boyal	**4**	**0**	**3**	**5**	**0**	**0**	**0**	**0**
Guadalupe I	0	0	0	0	0	0	0	0
Guadalupe II	1	0	1	1	0	0	0	0
Guadalupe III	4	0	0	0	0	0	0	0
Guadalupe IV	1	0	1	0	0	0	0	0
	**6**	**0**	**2**	**1**	**0**	**0**	**0**	**0**
Mengabril I	1	0	0	0	0	14	0	0
Mengabril II	1	0	0	0	0	0	0	82
Mengabril III	1	0	0	0	0	0	0	58
Mengabril IV	0	0	0	0	1	5	0	62
	**3**	**0**	**0**	**0**	**1**	**19**	**0**	**202**
Robledo I	1	0	0	0	1	0	0	0
Robledo II	1	0	1	0	0	0	1	0
	**2**	**0**	**1**	**0**	**1**	**0**	**1**	**0**
Serrezuela 3	**1**	**0**	**0**	**0**	**1**	**224**	**0**	**25**
Sierra de Fuentes I	1	0	0	0	0	0	0	2
Sierra de Fuentes II	0	0	0	0	0	1	2	1
Sierra de Fuentes III	2	0	0	0	0	1	1	4
Sierra de Fuentes IV	1	0	0	0	0	0	7	20
Sierra de Fuentes V	4	0	0	1	0	0	0	1
	**8**	**0**	**0**	**1**	**0**	**2**	**10**	**28**

Scats

Hunting area	Red fox	Stone marten	Eurasian badger	Common genet	Egyptian mongoose	European rabbit	Iberian hare	Red-legged partridge
Cabeza Redonda	**7**	**3**	**0**	**0**	**0**	**67**	**1**	**3**
Dehesa Boyal	**8**	**6**	**0**	**0**	**0**	**0**	**0**	**3**
Guadalupe I	1	7	0	0	0	0	0	4
Guadalupe II	4	6	0	0	0	0	0	0
Guadalupe III	5	6	0	0	0	0	0	37
Guadalupe IV	17	6	3	0	0	0	0	0
	**27**	**25**	**3**	**0**	**0**	**0**	**0**	**41**
Mengabril I	2	0	0	0	0	2	0	1
Mengabril II	1	0	0	0	0	2	0	4
Mengabril III	1	0	0	0	0	0	0	1
Mengabril IV	2	0	0	0	0	10	0	16
	**6**	**0**	**0**	**0**	**0**	**14**	**0**	**22**
Robledo I	3	1	0	1	0	1	0	2
Robledo II	7	1	0	1	0	0	3	4
	**10**	**2**	**0**	**2**	**0**	**1**	**3**	**6**
Serrezuela 3	**4**	**0**	**0**	**0**	**0**	**411**	**0**	**1**
Sierra de Fuentes I	3	0	0	0	0	1	2	0
Sierra de Fuentes II	4	0	0	0	0	0	0	0

Sierra de Fuentes III	4	0	0	0	0	22	40	12
Sierra de Fuentes IV	7	0	0	0	0	5	14	0
Sierra de Fuentes V	0	8	0	0	0	0	0	4
	**18**	**8**	**0**	**0**	**0**	**28**	**56**	**16**
**Sightings**								
Cabeza Redonda	**0**	**0**	**0**	**0**	**0**	**0**	**1**	**4**
Dehesa Boyal	**0**	**0**	**0**	**0**	**0**	**0**	**0**	**1**
Guadalupe I	1	7	0	0	0	0	0	1
Guadalupe II	0	0	0	0	0	0	0	0
Guadalupe III	0	0	0	0	0	0	0	0
Guadalupe IV	0	0	0	0	0	0	0	0
	**0**	**0**	**0**	**0**	**0**	**0**	**0**	**1**
Mengabril I	0	0	0	0	0	0	0	2
Mengabril II	0	0	0	0	0	0	0	43
Mengabril III	0	0	0	0	0	0	0	0
Mengabril IV	0	0	0	0	0	0	0	1
	**0**	**0**	**0**	**0**	**1**	**0**	**0**	**46**
Robledo I	0	0	0	0	0	0	1	0
Robledo II	1	0	0	0	2	0	3	8
	**1**	**0**	**0**	**0**	**2**	**0**	**4**	**8**
Serrezuela 3	**0**	**0**	**0**	**0**	**0**	**33**	**3**	**1**
Sierra de Fuentes I	0	0	0	0	0	0	0	0
Sierra de Fuentes II	0	0	0	0	0	0	0	0
Sierra de Fuentes III	0	0	0	0	0	1	0	0
Sierra de Fuentes IV	1	0	0	0	0	0	1	0
Sierra de Fuentes V	0	0	0	0	0	0	0	0
	**1**	**0**	**0**	**0**	**0**	**1**	**1**	**0**

Total relative abundance recorded across each hunting area is marked in bold
