# Supplementary material for: Social perception of mesocarnivores within hunting areas differs from actual species abundance
Source: PLoS One. 2023 Apr 26;18(4):e0283882. doi: 10.1371/journal.pone.0283882 (PMC10132647; doi:10.1371/journal.pone.0283882)
Supplement: Attachment — Submitted filename: Response to Reviewers.docx [file pone.0283882.s014.docx]

PLoS One, Manuscript PONE-D-22-29695

**Social perception of mesocarnivores within hunting areas differs from actual species abundance**

*1. Academic Editor Comments (if provided):

Most comments have been addressed. I recommend the following revisions:

Abstract:
I suggest replacing as follows:
"social perception and field data"
social perception and ecological field data.*

Text modified as suggested by the Editor (line 27 of the current version of the manuscript).

*2. "might help identifying"
might help identify*
Done (line 27 of the current version of the manuscript).

*3. "potential biases in human-carnivore conflicts"
potential biases in identifying and managing human-carnivore conflicts*

The sentence was rephrased as suggested by the editor (line 28 of the current version of the manuscript).

*4. "The main objective of this work is to analyse"
We analyzed*

The sentence was rephrased as suggested by the editor (line 28 of the current version of the manuscript).

*5. "perceived and the measured relative abundance"
perceived and field-measured relative abundance of carnivores*

The sentence was partially modified, given that field-measured abundance also includes small game species (line 29 of the current version of the manuscript).

*6. "mesocarnivores perceived abundance, for both stakeholders was quite different to actual species abundance"
mesocarnivore perceived abundances were generally different to actual species abundances*

The sentence was rephrased as suggested by the editor (lines 32-33 of the current version of the manuscript).

*7. "respondents’ ability to identify the species"
respondents’ ability to identify the carnivore species*

Done (lines 34-35 of the current version of the manuscript).

*8. "the need of increase people knowledge of species distribution and characteristics before adopting decisions when managing human-carnivore conflicts, especially for stakeholders that are directly involved in"
the need to increase people's knowledge on species distribution and ecological characteristics before adopting decisions when managing human-carnivore conflicts*

The sentence was modified (lines 35-37 of the current version of the manuscript).

*9. Keywords:
Add some keywords for methods used (e.g., track survey etc.)*

We removed the expressions ‘attitude towards carnivores’ and ‘field sampling’ from Keywords and replaced them with ‘track surveys’ and ‘scat surveys’ (line 40 of the current version of the manuscript).

*10. Table 2 caption:
"capacity of mesocarnivore identification"
I suggest replacing as follows:
ability to identify mesocarnivores*

The sentence was rephrased (lines 315-316 of the current version of the manuscript).

*11. Table 3:
Are the values in the table standardized by km2? They should be*

Values in Table 3 represent the relative abundance of mesocarnivores and small game species found in each 4-km^2^ sampling unit surveyed. However, following editor’s suggestion, we now provide, marked in bold, the total relative abundance standardized by hunting area surface in km^2^ (lines 375-377 of the current version of the manuscript).

*12. In your response to Reviewer 1, you state:
"On the other hand, Suárez-Tangil and Rodríguez (2021b) examined the influence of spatiotemporal factors on the performance of survey methods, concluding that i) after controlling for variations in mammal relative abundance or activity associated with landscape and season, the effect of spatio-temporal factors on the performance of detection methods was small; and ii) track surveys proved to be the most efficient and fastest method for detecting mammal species, being barely influenced by spatio-temporal factors. Both investigations were carried out in Mediterranean regions with similar land cover (agricultural landscapes and restricted natural vegetation areas), and comprised most of the target species studied in our manuscript. Therefore, we considered that their conclusions are applicable to our manuscript, and that the reliability of the field-sampling methods might not hinder the manuscript at all".

Please condense the information above and include it in the manuscript to highlight your assumption described above.*

We added the following sentence, written in lines 192-194 of the current version of the manuscript:

‘The regular performance and high efficiency of track and scat surveys have been proven in other Mediterranean regions with similar land covers’.
